# Evaluation Research for Intergenerational Programs: Rigorous Methods, Best Practices, and Challenges

**DOI:** 10.1093/geroni/igaa057.1764

**Published:** 2020-12-16

**Authors:** Skye Leedahl, Amy Eisenstein

## Abstract

Conducting evaluative research on intergenerational programs is key to understanding if they are functioning as intended. Research on program impact is also critical for prioritization and decision-making in an increasingly competitive market with many programming choices. Implementation studies can help researchers identify needed modifications for replication or introduction to new populations, which is particularly important as universities and communities work to become Age-Friendly. There is growing demand by educational entities (e.g., universities, high schools) and organizations that serve older adults (e.g., senior centers, adult day services, lifelong learning institutes, and residential programs) to identify rigorous methods that can be used to analyze outcomes for students and older adults who take part. This symposium focuses on evaluation methods for intergenerational programs and highlights diverse examples of how researchers have evaluated their programs. The presenters will discuss best practices and challenges to conducting research on these programs as well as the findings of the studies. The first paper will discuss the creation of an intergenerational contact measure. The second paper will describe how an intergenerational classroom was examined using data from instructors and students. The third paper will detail how a quasi-experimental design was used to examine outcomes for an intergenerational program on older adult participants. The fourth paper will discuss how data was gathered from various stakeholders to examine the impacts of an intergenerational classroom. Amy Eisenstein, Senior Program Officer at RRF Foundation for Aging will serve as the discussant. Intergenerational Learning, Research, and Community Engagement Interest Group Sponsored Symposium.

